# Is low pre-transplant parathyroid hormone a risk marker for cardiovascular disease in long-term follow-up of renal transplant recipients?

**DOI:** 10.1007/s10157-018-1543-9

**Published:** 2018-02-24

**Authors:** Elin Isaksson, Martin Almquist, Astrid Seeberger, Gunnar Sterner

**Affiliations:** 1 Department of Clinical Sciences, Faculty of Medicine, Lund University, Malmö, Sweden; 2 Department of Urology, Faculty of Medicine, Skåne University Hospital, Lund University, Jan Waldenströms gata 5, 20502 Malmö, SE Sweden; 3 Department of Clinical Sciences Lund, Skåne University Hospital, Lund University, Lund, Sweden; 4 Department of Surgery Section of Endocrine and Sarcoma Lund, Skane University Hospital, Lund, Sweden; 5 Department of Nephrology Huddinge, Faculty of Medicine, Karolinska University Hospital, Karolinska University, Solna, Sweden; 6 Department of Clinical Sciences, Skåne University Hospital, Lund University, Malmö, Sweden; 7 Department of Nephrology, Skane University Hospital, Malmö, Sweden

**Keywords:** Secondary hyperparathyroidism, Renal transplantation, Parathyroid hormone, Cardiovascular disease

## Abstract

**Background:**

Secondary hyperparathyroidism and altered levels of parathyroid hormone (PTH) are associated with vascular events in chronic kidney disease. After renal transplantation, this association is not clear. Pre-transplant parathyroidectomy (PTX) is common, but post-transplant data are scarce. We aimed to study the effect of PTH at the time of transplantation on risk of post-transplant vascular events in renal transplant recipients with and without pre-transplant PTX.

**Methods:**

258 patients from two Swedish transplant units were followed for 6 years. Separate analyses were made for patients with or without pre-transplant PTX. Patients with no pre-transplant PTX were stratified by quartiles of PTH at time of transplantation and patients with pre-transplant PTX were stratified by above and below median levels of PTH at time of transplantation. Hazard ratios for vascular events, mortality, and graft failure were calculated in adjusted Cox regression models.

**Results:**

In patients with no pre-transplant PTX, the lowest quartile of PTH at transplantation had a higher risk of cardiovascular events compared to quartile 3 with an adjusted hazard ratio (95% CI) of 2.63 (1.04–6.67). In patients with pre-transplant PTX, the group below median of PTH had a higher risk of cardiovascular events with an adjusted hazard ratio (95% CI) of 18.15 (1.62–203.82) compared to patients above median of PTH.

**Conclusion:**

Low levels of parathyroid hormone before transplantation were associated with increased risk of post-transplant vascular events both in patients with and without pre-transplant parathyroidectomy. Any conclusions on causal or direct effect of PTH on outcome cannot be drawn from this observational study.

## Introduction

Secondary hyperparathyroidism (sHPT) is common in chronic kidney disease and is associated with bone disease and vascular calcifications [1]. In spite of improved medical treatment for sHPT, surgical treatment with parathyroidectomy (PTX) is still often necessary [2]. In sHPT, the mineral metabolism is disturbed and many factors contribute to the associated morbidity. Levels of parathyroid hormone (PTH) are mainly used to grade the extent of sHPT and both high and low PTH have been associated with cardiovascular disease (CVD) in patients on maintenance dialysis [3–6]. Renal transplantation improves many of the underlying causes of sHPT and levels of PTH decrease after transplantation, even though sHPT persists in the majority of renal transplant recipients over the short as well as long term [7, 8]. Recent studies have shown no association between post-transplant PTH and risk of vascular events [9], but have shown an association with graft failure and mortality [10]. Data on pre-transplant PTH and outcomes are limited and few studies report whether patients have been treated with PTX before transplantation or not, which may influence the results. When evaluating patients for renal transplantation, the PTH level is one of many important parameters to include as sHPT can influence patient and graft survival. No upper PTH limit for renal transplantation has been defined, but hypercalcemia is generally not accepted. Our intention was to describe the relation between pre-transplant plasma PTH and long-term risk of incident cardiovascular disease after renal transplantation in patients with and without pre-transplant PTX.

## Subjects and methods

### Patient selection

We performed a retrospective study of patients above 18 years of age who underwent renal transplantation at Skåne University Hospital and Karolinska University Hospital between 1 January 2003 and 31 December 2005. The regional ethical review board approved the study on the condition that patients alive at follow-up (1st of February 2011) gave informed, written, consent.

### Search of medical records

We manually searched patients’ medical records using a pre-specified form. We defined the baseline as the date of transplantation and the endpoint as 6 year post-transplantation, death or graft failure, whichever occurred first. Demographic data and years on dialysis treatment were obtained as well as any history of parathyroidectomy (PTX), ongoing treatment with calcimimetics, diabetes, prevalent cardiovascular disease, treatment of hypertension, hyperlipidemia, and hyperuricemia was noted as well as any history of smoking and type of graft: from living donor or deceased donor. During the observation period, any new, incident vascular event (myocardial infarction, coronary revascularization procedure, cerebral infarction, transient ischemic attack, peripheral vessel revascularization, amputation, or death from cardiovascular disease) was noted from medical records. Only the first vascular event was recorded. Immunosuppressive treatment was noted as well as all treatment of mineral metabolism such as calcimimetics, vitamin D, calcium supplements, phosphate binders, and parathyroidectomy after transplantation.

### Laboratory analyses

Laboratory data of PTH, phosphate, creatinine, calcium, albumin, triglycerides, low-density lipoprotein (LDL), high-density lipoprotein (HDL), uric acid, and C-reactive protein (CRP) were collected prior to the date of transplantation. PTH, phosphate, creatinine, calcium, and albumin were thereafter collected yearly after transplantation. Plasma phosphate (normal range 0.7–1.5 mmol/L), calcium (normal range 2.15–2.50 mmol/L), albumin (normal range 18–40 years 36–48 g/L, 41–70 years 36–45 g/L), creatinine (normal range 45–90 µmol/L female, 60–105 µmol/L male), LDL mmol/L (normal range 1.4–4.7 mmol/L), HDL mmol/L (normal range female 1.0-2.7 male 0.8–2.1 mmol/L), triglycerides mmol/L (normal range 0.4–2.6 mmol/L), uric acid µmol/L (normal range female 155–350, male 230–480 µmol/L), and CRP mg/L (normal range < 3 mg/L) were determined using routine methods. Albumin-corrected calcium was calculated by the following formula Ca-corr = P − Ca + (0.02 × (40 − P-albumin)). Levels of PTH (normal range 1.6–6.9 pmol/L) were determined by a two-site chemiluminescent immunometric assay and were analyzed either using Cobas e411/Elecsys, Roche or Immulite 2000, Siemens. Since the different PTH assays were not entirely comparable, we used an adjusting formula (Cobas = 0.7439 × Immulite + 0.7351) defined at the Department of Clinical Chemistry at Skane University Hospital. The glomerular filtration rate was estimated by a creatinine-based equation (Lund–Malmö glomerular filtration rate prediction equation without body weight measure) [11] and results given as mL/min/1.73 m^2^. Plasma creatinine assays employed calibration traceable to a common reference material and a zero-point calibrator, both issued as part of the NORIP project [12, 13]. Length and weight were obtained at the time of discharge from the transplantation unit and we calculated body mass index (BMI) by the following formula: weight (kg)/(length (m))^2^.

### Statistical analyses

We divided the patients into two groups: patients with no pre-transplant PTX and patients with pre-transplant PTX. Patients with no PTX were stratified by quartiles of PTH levels at the time of transplantation. Due to a small patient number, patients with PTX were stratified above and below median PTH levels at the time of transplantation. Differences in laboratory values between groups were calculated using a Kruskal–Wallis test or a Mann–Whitney test where appropriate. Cardiovascular event free survival was estimated by Kaplan–Meier curves. Cox’s regression models were applied to evaluate the risk for cardiovascular events, using quartile 3 of PTH as the reference category in non-PTX patients and above median as the reference category in PTX patients. Adjustments were made for the following factors: age, gender, diabetes, a history of CVD, BMI at time of transplantation, and years on dialysis before transplantation. These factors have been shown to predict vascular morbidity and death in renal transplant recipients [14]. Gender, diabetes, and a history of CVD were adjusted as categorical variables using male gender, no diabetes, and no history of cardiovascular disease as reference categories. The same model was used to calculate hazard ratios for mortality and graft failure in non-PTX patients. As a sensitivity analysis, we further adjusted for levels of uric acid (continuous), treatment with alfacalcidol after transplantation (categorical), and treatment with cholecalciferol after transplantation (categorical), in addition to the original model. In the Cox model, patients were followed for 6 years (starting at the date of RT). Patients were censored when an event occurred, at graft failure, or at death, whichever occurred first. All statistical analyses were performed using SPSS 22.0 (SPSS Inc., Chicago, Ill., USA). Statistical significance was considered with a *p* value of < 0.05.

## Results

A total of 258 patients were included in the study, whereof 36 were parathyroidectomized before transplantation. Baseline demographic data of patients in the different groups are presented in Table 1. During the follow-up, there were a total of 55 incident vascular events. Twenty-five patients suffered from myocardial infarction, 15 from peripheral vascular events, and 15 from stroke. Overall mortality was 10% (n = 26) at the endpoint. Of these, 14 were caused by CVD, seven by malignancy, and five had other causes. The number of patients with pre-existing cardiovascular disease, a history of smoking and hypertension as well as anti-hypertensive treatment did not differ significantly between groups and the numbers are summarized Table 1. The majority of patients were treated with triple immunosuppressive treatment which consisted of prednisolone and either tacrolimus and mycophenolate mofetil (MMF) (60%), cyclosporin A and MMF (15%), or tacrolimus and azathioprin (15%). Ten percent received other immunosuppressive drugs. Median (interquartile range, IQR) follow-up for cardiovascular events was 72 (56–72) months and for graft failure and overall mortality 72 (72–72).

**Table 1:** Baseline characteristics of Swedish renal transplant recipients (*n* = 258) with and without parathyroidectomy before transplantation, stratified by levels of PTH at time of transplantation

Factor	No PTX before transplantation (*n* = 222)					PTX before transplantation (*n* = 36)		
	PTH quartile 1	PTH quartile 2	PTH quartile 3	PTH quartile 4	*p* value	PTH Below median	PTH Above median	*p* value
Gender								
Male	44 (77)	38 (69)	37 (65)	38 (72)	0.537^a^	10 (56)	11 (61)	0.735^a^
Female	13 (23)	17 (31)	20 (31)	15 (28)		8 (44)	7 (39)	
Median age in years (range)	50 (19–71)	55 (31–72)	52 (22–73)	49 (19–70)	0.046^b^	50 (33–68)	57 (18–73)	0.205^c^
Original kidney disease								
Glomerulonephritis	23 (40)	16 (29)	19 (33)	20 (38)	0.486^a^	7 (40)	10 (58)	0.562^a^
Diabetes	7 (12)	9 (16)	10 (18)	6 (11)		2 (11)	1 (5)	
Vasculitis	2 (4)	4 (7)	4 (7)	3 (6)		1 (5)	2 (11)	
Hereditary	13 (23)	14 (26)	10 (18)	8 (15)		5 (28)	1 (5)	
Congenital	4 (7)	0 (0)	3 (5)	7 (13)		2 (11)	2 (11)	
Nephrosclerosis	1 (2)	6 (11)	5 (9)	2 (4)		1 (5)	1 (5)	
Other/unknown	7 (12)	6 (11)	6 (10)	7 (13)		0 (0)	1 (5)	
Living donor graft	29 (51)	19 (35)	20 (35)	25 (47)	0.187^a^	6 (33)	5 (28)	0.717^a^
First transplant	54 (95)	51 (93)	50 (88)	39 (74)	0.004^a^	11 (61)	9 (50)	0.502^a^
Pre-TX diabetes	17 (30)	14 (26)	17 (30)	13 (24)	0.883^a^	15 (83)	7 (39)	0.137^a^
Years in dialysis	1.7 (0.75–3.0)	2.0 (1.0–3.0)	2.0 (1.0–4.0)	2.5 (1.0-4.5)	0.264^b^	4.2 (1.9–7.5)	4.5 (2.4–8.5)	0.657^c^
Type of dialysis								
HD	29 (51)	32 (58)	40 (70)	35 (66)	0.472^a^	13 (78)	11 (61)	0.461^a^
PD	24 (42)	20 (36)	13 (23)	12 (23)		4 (22)	7 (39)	
None	4 (7)	3 (6)	4 (7)	6 (11)		1 (5)		
Previous CVD	9 (16)	17 (31)	16 (18)	12 (23)	0.254^a^	3 (17)	4 (22)	0.674^a^
Previous smoker	16 (29)	17 (31)	17 (30)	10 (20)	0.544^a^	6 (33)	5 (28)	0.717^a^
Hypertension	40 (71)	36 (66)	40 (70)	29 (57)	0.378^a^	11 (61)	8 (44)	0.317^a^
Hyperuricemia	4 (8)	5 (9)	3 (6)	5 (10)	0.824^a^	1 (6)	4 (24)	0.129^a^
Treatment with statins	22 (42)	21 (40)	21 (39)	14 (29)	0.508^a^	9 (50)	8 (47)	0.862^a^
Treatment with beta-blockers	27 (52)	32 (60)	32 (59)	27 (55)	0.663^a^	9 (50)	7 (41)	0.600^a^
Treatment with ACEi or ARB’s	22 (43)	18 (34)	22 (42)	23 (47)	0.592^a^	6 (33)	5 (29)	0.803^a^
Treatment with calcium channel blockers	35 (67)	24 (44)	22 (41)	16 (33)	0.003^a^	8 (44)	4 (24)	0.193^a^
Laboratory measurements before transplantation								
Uric acid µmol/L	295 (238–370)	309 (235–391)	352 (261–444)	430 (307–492)	0.009^b^	375 (317–461)	318 (257–390)	0.223^c^
CRP mg/L	1 (1–3)	3 (1–9)	1 (1–11)	1 (1–7)	0.075^b^	1 (1–4)	2 (1–10)	0.493^c^
Albumin g/L	33 (31–37)	33 (29–36)	33 (29–37)	31 (28–35)	0.091^b^	34 (27–38)	33 (31–37)	0.938^c^
Body mass index kg/m^2^	24 (21–27)	25 (21–27)	24 (22–27)	26 (22–28)	0.441^b^	25 (21–29)	25 (24–27)	0.988^c^

Values are numbers (%) or medians (interquartile range) where appropriate

*PTX* parathyroidectomy, *PTH* parathyroid hormone, *TX* transplantation, *HD* hemodialysis, *PD* peritoneal dialysis, *CVD* cardiovascular disease, *ACEi* angiotensin converting enzyme inhibitor, *ARB* angiotensin receptor blocker, *CRP* C-reactive protein

^a^Chi^2^ test

^b^Kruskal–Wallis test

^c^Mann–Whitney test

### Laboratory analyses

Levels of PTH, phosphate, calcium, and eGFR at start, 2, 4, and 6 years after transplantation in strata are described in Table 2. PTH decreased from preoperative values to 2 years after transplantation but remained fairly stable beyond year 2 through follow-up in non-PTX groups. Levels of albumin-corrected calcium were higher in groups with higher PTH in non-PTX patients through follow-up. Levels of uric acid differed significantly between groups of non-PTX patients. Patients in the highest quartile of PTH had higher uric acid levels compared to patients in the lower quartiles of PTH. In PTX patients, PTH levels were stable after transplantation and levels of albumin-corrected calcium were lower in the group with lower PTH at the start, 2 and 4 years after transplantation. In the same group, phosphate was higher at 2, 4, and 6 years compared to patients with PTH above median. Pre-transplant levels of CRP, albumin, and BMI did not differ between groups (Table 1). There were no significant differences between levels of plasma lipids before transplantation.

**Table 2:** Six-year measures of PTH, calcium, phosphate, and eGFR in Swedish renal transplant recipients with and without parathyroidectomy before transplantation, stratified by levels of PTH at time of transplantation (*n* = 258)

Factor	No PTX (*n* = 222)					PTX (*n* = 36)		
	PTH quartile 1	PTH quartile 2	PTH quartile 3	PTH quartile 4	*p* value^a^	PTH below median	PTH above median	*p* value^b^
p-PTH median, IQR (pmol/L)								
Start	6.5 (4.7–8.5)	12.4 (10.7–14.2)	21.0 (18.6–24.1)	44.0 (31.2–68.1)	< 0.001	1.5 (0.7–3.8)	14.0 (9.4–18.0)	< 0.001
2 years	7.0 (5.6–9.8)	10.2 (6.8–13.0)	11.0 (7.8–18.8)	14.6 (8.8–20.9)	< 0.001	2.1 (0.7–4.6)	8.4 (5.1–13.4)	< 0.001
4 ^years^	7.1 (4.9–10.3)	10.0 (6.7–14.8)	11.2 (7.6–18.8)	14.3 (9.3–21.2)	< 0.001	2.2 (0.8-4.0)	9.6 (7.2–14.0)	< 0.001
6 years	7.5 (5.6–12.7)	10.8 (8.3–14.7)	13.4 (8.1–18.1)	14.0 (10.0-19.1)	0.001	1.9 (0.9–4.4)	8.3 (6.1–13.3)	< 0.001
p-Calcium adjusted for albumin median, IQR (mmol/L)								
Start	2.56 (2.44–2.67)	2.48 (2.38–2.65)	2.54 (2.38–2.70)	2.52 (2.39–2.76)	0.794	2.47 (2.20–2.58)	2.47 (2.26–2.65)	0.410
2 years	2.38 (2.34–2.51)	2.46 (2.37–2.55)	2.47 (2.39–2.55)	2.46 (2.39–2.58)	0.003	2.22 (2.07–2.32)	2.40 (2.34–2.58)	0.002
4 years	2.38 (2.31–2.46)	2.44 (2.41–2.55)	2.47 (2.37–2.62)	2.54 (2.39–2.63)	0.001	2.31 (2.19–2.40)	2.39 (2.21–2.54)	0.222
6 years	2.39 (2.34–2.50)	2.45 (2.37–2.50)	2.45 (2.35–2.54)	2.48 (2.41–2.54)	0.116	2.22 (2.03–2.30)	2.38 (2.27–2.47)	0.057
p-Phosphate median, IQR (mmol/L)								
Start	1.54 (1.11–1.89)	1.64 (1.20–2.05)	1.50 (1.11-2.00)	2.05 (1.56–2.30)	< 0.001	1.41 (1.00–2.00)	1.50 (1.10–1.98)	0.895
2 years	1.03 (0.95–1.20)	0.95 (0.81–1.10)	0.98 (0.88–1.10)	0.90 (0.78–1.10)	0.007	1.20 (1.10–1.49)	1.03 (0.89–1.17)	0.002
4 years	1.07 (0.87–1.16)	1.00 (0.90–1.10)	0.99 (0.88–1.10)	0.91 (0.76–1.05)	0.014	1.32 (0.94–1.48)	0.99 (0.83–1.21)	0.036
6 years	1.00 (0.90–1.20)	1.00 (0.89–1.19)	0.96 (0.85–1.10)	0.94 (0.81–1.13)	0.174	1.25 (1.10–1.72)	0.95 (0.83–1.14)	0.003
eGFR median, IQR (ml/min/1.73 m^2^)								
Start	8 (7–10)	8 (6–10)	9 (7–11)	8 (6–10)	0.314	8 (6–10)	8 (6–9)	0.950
2 years	66 (56–80)	61 (35–80)	59 (34–77)	56 (32–82)	0.423	56 (32–67)	63 (47–78)	0.181
4 years	65 (48–81)	61 (42–83)	62 (35–83)	51 (31–81)	0.262	57 (36–71)	64 (37–76)	0.397
6 years	69 (43–87)	58 (29–77)	62 (30–73)	53 (30–77)	0.269	59 (35–75)	71 (35–85)	0.376

*PTH* parathyroid hormone, *eGFR* estimated glomerular filtration rate, *IQR* interquartile range

^a^Kruskal–Wallis test between quartiles of PTH at time of transplantation in patients with no pre-transplant PTX

^b^Mann–Whitney test between above and below median of PTH at time of transplantation in patients with pre-transplant PTX

### Post-transplant treatment for sHPT

The number of patients treated with PTX and calcimimeticum after transplantation was numerically but not significantly higher in the non-PTX transplanted patients during the 6 years of follow-up. In the group of patients who underwent PTX before transplantation, two out of 18 were treated with calcimimeticum after transplantation. Treatment with alfacalcidol was more common in patients in the higher quartiles of pre-transplant PTH and treatment with cholecalciferol was more common in patients with lower quartiles of pre-transplant PTH. All post-transplant sHPT treatments are summarized in Table 3.

**Table 3:** Post-transplant hyperparathyroidism treatment and hypercalcemia in Swedish renal transplant recipients with and without parathyroidectomy before transplantation, stratified by levels of PTH at time of transplantation (*n* = 258)

	No PTX before transplantation					PTX before transplantation		
Factor	PTH quartile 1	PTH quartile 2	PTH quartile 3	PTH quartile 4	*p* value	PTH Below median	PTH Above median	*p* value
PTX								
Yes	1 (2)	0 (0)	3 (5)	5 (9)	0.087	0 (0)	0 (0)	n.a
Calcimimetics								
Yes	0 (0)	5 (9)	6 (11)	6 (11)	0.064	0 (0)	2 (11)	0.146
Alfacalcidol								
Yes	16 (29)	20 (39)	27 (50)	30 (61)	0.007	9 (60)	7 (39)	0.227
Cholecalciferol								
Yes	29 (53)	21 (40)	18 (33)	8 (16)	0.001	6 (40)	10 (56)	0.373
Phosphate binders								
Yes	2 (4)	2 (4)	1 (2)	2 (4)	0.919	2 (11)	1 (6)	0.546
Calcium supplements								
Yes	34 (62)	28 (54)	22 (41)	20 (41)	0.076	15 (100)	13 (72)	0.027
Hypercalcemia year 1								
Yes	13 (25)	22 (44)	24 (45)	33 (65)	0.001	12 (80)	8 (50)	0.081
No	40 (75)	28 (56)	29 (55)	18 (35)		3 (20)	8 (50)	
Hypercalcemia year 3								
Yes	6 (13)	17 (37)	12 (25)	21 (48)	0.002	3 (23)	3 (20)	0.843
No	40 (87)	29 (63)	36 (75)	23 (52)		10 (77)	12 (80)	
Hypercalcemia year 6								
Yes	10 (23)	9 (23)	15 (35)	14 (37)	0.319	2 (17)	3 (21)	0.759
No	34 (77)	31 (78)	28 (65)	29 (63)		10 (83)	11 (79)	

### Outcome in renal transplant recipients without PTX before transplantation

**Fig. 1:**
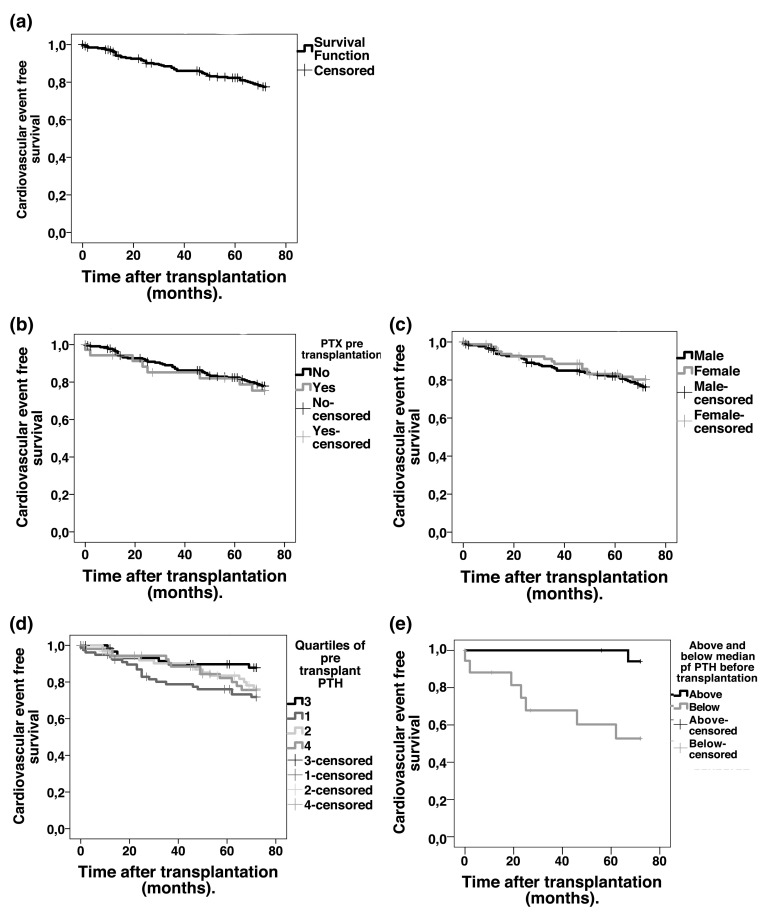
Kaplan–Meier cardiovascular event free survival curves in 258 Swedish patients undergoing renal transplantation censoring if event, death or graft loss, mean follow-up 6 years. **a** All patients, **b** all patients divided by parathyroidectomy (PTX) or not prior to transplantation. **c** All patients divided by gender. **d** Patients with no PTX before transplantation divided by quartiles of pre-transplant levels of parathyroid hormone (PTH) (*n* = 222). **e** patients with pre-transplant PTX divided by above and below median of pre-transplant PTH (*n* = 36)

Hazard ratios (95% CI) of incident vascular events across strata in patients with no pre-transplant PTX are summarized in Table 4. The lowest quartile of PTH showed an increased risk of vascular events with a hazard ratio (95% CI) of 2.63 (1.04–6.67) compared to reference. Age at the time of transplantation, a history of cardiovascular disease, and diabetes at the time of transplantation were all associated with outcome. When adjusting for levels of uric acid before transplantation or treatment with alfacalcidol or cholecalciferol after transplantation, the confidence intervals were wider, but hazard ratios for cardiovascular events in quartiles of PTH were similar (data not shown). Hazard ratios (95% CI) of overall mortality across strata of pre-transplant PTH did not differ significantly to reference values in the full adjusted Cox regression model and were 1.47 (0.41–5.33) in quartile 1, 1.26 (0.38–4.15) in quartile 2 and 1.47 (0.42–5.16) in quartile 4 using quartile 3 as a reference. Only a history of CVD remained significantly associated with overall mortality in the full model with a hazard ratio (95% CI) of 4.36 (1.73–10.99) compared to patients with no history of CVD. Quartiles of pre-transplant PTH were not associated with graft failure in the same Cox regression model. Hazard ratios (95% CI) were 0.66 (0.15–2.79) in quartile 1, 1.22 (0.36–4.10) in quartile 2 and 1.77 (0.59–5.32) in quartile 4 using quartile 3 as a reference. Only years in dialysis before transplantation remained significantly associated with graft failure in the full model with a hazard ratio (95% CI) of 1.21 (1.03–1.42) for each extra year spent in dialysis before transplantation. Kaplan–Meier curves depicting cardiovascular event free survival compared by gender, PTX or not before transplantation, and by different levels of PTH before transplantation are shown in Fig. 1.

**Table 4:** Hazard ratio (95% CI) of vascular events in renal transplant recipients with no prior PTX followed for 6 year post-transplantation, stratified in quartiles of levels of PTH at time of renal transplantation (*n* = 222)

Factor	Number of patients *N* (%)	Number of events *N* (%)	Crude HR (95% CI)	Adjusted HR (95% CI)
Quartiles of PTH at time of transplantation pmol/L				
1	57 (26)	14 (25)	2.01 (0.81–4.99)	2.63 (1.04–6.67)
2	55 (24)	13 (24)	1.94 (0.78–4.87)	2.02 (0.80–5.12)
3	57 (26)	7 (12)	1.00	1.00
4	53 (24)	12 (23)	1.85 (0.73–4.70)	2.12 (0.81–5.57)
Age			1.05 (1.02–1.08)	1.04 (1.01–1.08)
Gender				
Male	157 (71)	35 (22)	1.00	1.00
Female	65 (29)	12 (18)	0.82 (0.43–1.58)	1.08 (0.52–2.25)
Years in dialysis before transplantation			1.16 (1.04–1.30)	1.15 (1.00-1.31)
History of CVD				
No	168 (76)	21 (40)	1.00	1.00
Yes	54 (24)	26 (15)	3.27 (1.84–5.82)	1.97 (1.02–3.80)
Diabetes				
No	161 (72)	25 (40)	1.00	1.00
Yes	61 (28)	22 (13)	3.40 (1.92–6.04)	2.57 (1.36–4.83)
Body Mass Index at time of transplantation kg/m^2^			1.01 (9.94–1.08)	0.96 (0.89–1.04)

*PTX* parathyroidectomy, *PTH* parathyroid hormone, *CVD* cardiovascular disease

### Outcome in renal transplant recipients with PTX before transplantation

Hazard ratios (95% CI) of incident vascular events across strata in patients with no pre-transplant PTX are summarized in Table 5. In patients with pre-transplant PTX, the group below median of PTH had a higher risk of vascular disease with an adjusted hazard ratio (95% CI) of 18.15 (1.62–203.82) compared to patients above the median of PTH. A history of CVD was associated with incident vascular events, but pre-transplant diabetes was not. There were too few events to obtain any statistics on overall mortality or graft failures in patients with a pre-transplant PTX, which is why this has not been done.

**Table 5:** Hazard ratio (95% CI) of vascular events in renal transplant recipients with prior PTX followed for 6 year post-transplantation, stratified in groups based on levels of PTH at time of renal transplantation (*n* = 36)

Factor	Number of patients *N* (%)	Number of events *N* (%)	Crude HR (95% CI)	Adjusted HR (95% CI)
Below and above PTH 6.6 pmol/L				
Below	18 (50)	7 (39)	11.51 (1.40-94.33)	18.15 (1.62-203.82)
Above	18 (50)	4 (22)	1.00	1.00
Age			1.10 (1.01–1.19)	1.09 (1.00-1.17)
Gender				
Male	21 (58)	5 (24)	1.00	1.00
Female	15 (42)	3 (20)	0.77 (0.18–3.24)	0.41 (0.06–2.78)
Years in dialysis before transplantation			0.97 (0.83–1.15)	0.88 (0.75–1.04)
History of CVD				
No	29 (81)	6 (21)	1.00	1.00
Yes	7 (19)	2 (29)	1.25 (0.25–6.19)	1.18 (1.10–13.47)
Diabetes				
No	22 (61)	6 (23)	1.00	1.00
Yes	14 (39)	2 (20)	0.73 (0.15–3.64)	0.29 (0.02–3.72)
BMI at time of transplantation kg/m^2^			0.92 (0.76–1.12)	0.80 (0.60–1.08)

*PTX* parathyroidectomy, *PTH* parathyroid hormone, *CVD* cardiovascular disease, *BMI* body mass index

## Discussion

In this observational study our main findings were that low PTH at the time of transplantation was associated with a higher risk of post-transplant cardiovascular events. Surprisingly, patients in the highest quartile of p-PTH before renal transplantation did not seem to suffer higher risk of cardiovascular events, mortality or graft failure during 6 years of follow-up. This is contrary to a large study in patients in dialysis [6], where high PTH was associated with higher risk of mortality and cardiovascular-related hospitalization. That study differs from the present study in that it did not include patients with a renal transplant. Furthermore, the higher risk for cardiovascular hospitalization was only seen in patients with PTH above 600 pg/mL (approximately 63.7 pmol/L) in that study, which is above the levels of the highest quartile in our study, making comparisons between studies difficult. Data on pre-transplant PTH and post-transplant outcomes are scarce but Roodnat et. al. [15] found a significant positive correlation between pre-transplant levels of PTH and graft failure. We found no such association; during 6 years of follow-up, GFR levels tended to be stable in all PTH groups. There are studies on post-transplant PTH levels and cardiovascular risk. Marcen et al. [16] performed a study on 331 renal transplant recipients followed for 7 years and found no correlation between PTH at 1 month post-transplantation and cardiovascular events. Similarly, Pihlstrom et al. [10] studied a large cohort of renal transplant recipients (*n* = 1840) for a mean of 7 years and found no correlation between post-transplant PTH and CVD. Bleskestad et al. [17] studied 438 renal transplant recipients with preserved graft function and found an increased risk of a combined endpoint of CVD, graft failure, and death correlated with quartiles of 10 week post-RT PTH. The risk of CVD exclusively is not reported in the study. However, the patients in our study with the highest risk of vascular disease after transplantation had PTH levels at the time of transplantation below 9.5 pmol/L (no PTX pre-transplantation) and 6.6 pmol/L (PTX pre-transplantation), which is markedly low. The median level of PTH in patients with pre-transplant PTX was 1.5 pmol/L. Prior studies in patients with ongoing dialysis show that PTH levels below 65 pg/mL (approximately 6.9 pmol/L) can predict mortality and vascular outcomes [4, 5, 18] which corresponds to our findings. For patients on dialysis, the higher risk of mortality and vascular outcomes in patients with low levels of PTH has been explained by older age, malnutrition, and poor protein intake [4]. Lee et al. found that patients on dialysis with PTH levels below 65 pg/mL had a higher risk of vascular events and mortality compared to patients with PTH above 65 pg/mL and suggested that this was driven by vascular calcifications. This was supported by a higher progression rate of aortic arch calcification scores in the group with low PTH [5]. In our study, patients with low PTH were not of older age and indirect measures of malnutrition such as levels of albumin and BMI did not differ between groups. Another possible explanation for the higher risk of vascular disease in patients with low PTH is post-transplant bone disease [19]. Bone biopsies early after renal transplantation show reduced activity of osteoblasts [20] and patients with low PTH pre-transplant show lower post-transplant osteoblastic activity [21]. This reduced cellularity and low bone turnover can develop into an adynamic state of the bone which diminishes the ability of the bone to buffer elevations in blood levels of phosphorus and calcium, which in turn leads to calcifications of soft tissue and vessels [22] and thereby a higher risk of vascular morbidity. Hypercalcemia was more frequent in the groups with higher PTH and no pre-transplant PTX during follow-up. This is probably caused by PTH mediated calcium release from the skeleton [23].

Distribution of traditional cardiovascular risk factors such as hypertension, smoking and lipid status before transplantation did not differ between groups. Low and high levels of uric acid have been associated with cardiovascular disease and mortality in dialysis patients [24], and in our study, levels of uric acid were significantly higher in the highest quartile of PTH, but did not differ between quartiles 1, 2, and 3. Adjusting for levels of uric acid did not alter the association between PTH and cardiovascular disease why this cannot explain the high risk of CVD in the lowest quartile of PTH.

Treatment for post-transplant sHPT differed between groups. Patients with no pre-transplant PTX with higher PTH were more often treated with active vitamin D (alfacalcidol). This may have influenced the results, since treatment with active vitamin D has been associated with reduced mortality in dialysis patients [25]. Patients with no pre-transplant PTX with lower levels of PTH received more cholecalciferol compared to patients with higher PTH. Patients with higher PTH might not have been given cholecalciferol, since cholecalciferol treatments were combined with calcium supplements in most cases and patients with higher PTH had higher calcium levels during follow-up. However, including either alfacalcidol or cholecalciferol treatment after transplantation did not affect hazard ratios for cardiovascular disease between quartiles of PTH.

The immunosuppressive treatment protocols at the institutions in the present study have undergone only minor changes since 2003–2005, mainly with reduction of steroid dose. We deem it unlikely that the hyperparathyroid state has been affected by this change.

Our findings that patients with a pre-transplant PTX and low pre-transplant levels of PTH suffer from an increased risk of post-transplant vascular disease is of clinical importance, especially since there is an ongoing debate about whether to perform PTX before or after transplantation [26].

## Limitations

Patient numbers were relatively small, which could influence the results. However, we included two centers in Sweden, which hopefully makes the results more accurate for the overall Swedish population of renal transplant recipients. Levels of PTH were analyzed with two different techniques (40 patients had their PTH levels analyzed with Immulite 2000). This is a methodological problem that is hard to assess in the setting of a multicenter observational study and it can potentially influence the results. We made efforts to correct the issue, which is shown in our methods. Patients were selected during 2003–2005 and outcomes in renal transplant patients might differ from today why this must be taken into consideration when interpreting the results. Our study is a retrospective analysis and consequently burdened with some possible sources of bias.

## Conclusion

Low (less than 6.9 pmol/L) levels of parathyroid hormone before transplantation were associated with a higher risk of post-transplant vascular events both in patients with and without pre-transplant parathyroidectomy. Any conclusions on causal or direct effect of PTH on outcome cannot be drawn from this observational study.
